# Digital Pathology Displays Under Pressure: Benchmarking Performance Across Market Grades

**DOI:** 10.1007/s10278-025-01452-3

**Published:** 2025-02-26

**Authors:** Stefano Marletta, Alessandro Caputo, Gabriele Guidi, Liron Pantanowitz, Fabio Pagni, Iacopo Bavieri, Vincenzo L’Imperio, Matteo Brunelli, Angelo Paolo Dei Tos, Albino Eccher

**Affiliations:** 1 Division of Pathology, Humanitas Istituto Clinico Catanese, Catania, Italy; 2 Department of Diagnostics and Public Health, Section of Pathology, University of Verona, Verona, Italy; 3 Pathology Department, University Hospital “San Giovanni Di Dio E Ruggi d’Aragona”, Salerno, Italy; 4 Medical Physics Unit, University Hospital of Modena, Modena, Italy; 5 Department of Pathology, University of Pittsburgh, Pittsburgh, PA USA; 6 Department of Medicine and Surgery, Pathology, IRCCS Fondazione San Gerardo Dei Tintori, University of Milano-Bicocca, Monza, Italy; 7 Surgical Pathology and Cytopathology Unit, Department of Medicine‑DIMED, University of Padua School of Medicine, Padua, Italy; 8 Department of Medical and Sciences for Children and Adults, University of Modena and Reggio Emilia, University Hospital of Modena, Modena, Italy

**Keywords:** Digital pathology, Display, Luminance, Color, Performance, Computer monitor

## Abstract

Digital pathology (DP) has transformed the practice of pathology by digitizing pathology glass slides, thereby enhancing diagnostic capabilities. In contrast to radiology, studies comparing the efficiency of DP monitors are limited. This work used a stress test that simulated DP sign-out in practice to evaluate the performance of medical-grade (MG) and consumer off-the-shelf (COTS) displays. Four displays, including three MG and one COTS, were assessed for luminance, contrast ratio, accuracy, and image uniformity. Key metrics, such as luminance uniformity and maximum brightness, were evaluated during a 1-month period that simulated use to reflect an 8-h work day. MG displays outperformed COTS in critical parameters, even though consumer displays were satisfactory for diagnostic purposes. Image uniformity exhibited the most significant variations, with deterioration noted over 2.5% for all displays during the test period. This study compared different types of displays for DP and highlights the importance of regular calibration for maintaining display performance when using DP. Further research is recommended to define validation protocols, including the impact of display aging on DP accuracy.

**Supplementary Information:**

The online version contains supplementary material available at 10.1007/s10278-025-01452-3.

## Introduction

Digital pathology (DP) has revolutionized the practice of pathology. This disruptive technology involves scanning conventional glass slides, either partially or entirely (“whole slide imaging,” WSI), to obtain digital images that can be remotely visualized and manipulated on a monitor [1]. The advantages of DP include archiving and easy access, ease of navigation (such as zooming and rotation of images), annotation capabilities, image analysis, and the ability to share digital slides for remote teleconsultation (telepathology). As a result, DP has increasingly gained adoption, especially with the continuous development of improved scanners and monitors for diagnostic and research purposes [2]. Published evidence keeps broadening the application of DP, often in conjunction with artificial intelligence (AI) tools. These advancements extend even to small portable devices such as tablets and smartphones [3, 4], which offer a more affordable solution for resource-limited institutions to embrace DP.

According to their technical characteristics, computer displays fall into three main categories: (i) medical grade (MG), built for multiyear use and to provide a uniform experience to users; (ii) consumer off-the-shelf (COTS), general-purpose devices utilized by everyone from physicians to office workers and routine everyday home use; and (iii) professional-grade (PG), intermediate performance tools, with lower accuracy than MG monitors but higher than COTS for specific parameters (i.e., color depth, color gamut, brightness, and contrast ratio) [5]. Nevertheless, for medical imaging, internationally recognized WSI guidelines for the purpose of rendering a primary pathology diagnosis based on a digital slide [6], recommend employing mostly MG displays with specific standardized features [5, 7]. Among these technical specifications is luminance, which refers to the objective amount of light generated or reflected by a given area, measured in candela per square meter (cd/m^2^) [8]. The concept of luminance is closely linked with brightness and illuminance. The former is a subjective impression of light intensity, expressed in percentage, which can be influenced by many external factors apart from pure luminance. Light from the surrounding environment represents ambient illuminance, measured in lux (lx) [9].

Both brightness and illuminance can significantly influence the optimal luminance of a display and, therefore, the user’s perspective of image quality. Too high a level of ambient illuminance will have a negative impact on image quality and detection of subtle differences; conversely, if the illuminance exceeds the luminance generated from the panel’s surface, the viewer will face difficulties appreciating the contents of the image being displayed; conversely, if the ambient illuminance is too dark compared to the monitor, this can cause the image to appear too bright. Thus, in order to balance the effects of background illumination, some monitors are built with antireflective properties and incorporated within optical sensors, adjusting the display’s luminance according to the ambient light [5]. However, not all marketed MG monitors have such optical sensors and, more importantly, most of these devices tend to lose their ability to reach peak luminance as they age. Additionally, AAPM reports indicate adequate calibration, if at all ensure consistent luminance levels and compliance with standards [9]. Hence, constant device calibration of luminance and other technical display properties (e.g., color depth and gamut, contrast, adaptation time) is warranted to ensure optimal device quality is maintained. On this topic, the radiology literature advocates periodic monitoring to check a panel's performance, which should be conducted at least monthly for each monitor employed for primary diagnostic interpretation, according to specific accredited quality control (QC) programs [10]. Information regarding which parameters warrant periodic QC checks for DP monitors, as well as the frequency for undertaking such calibration tests, is lacking. However, published evidence highlights that better quality and strict display calibration often ensure higher performances, at least in terms of reading time [11–13].

Despite initial skepticism from the pathology community [14], in recent years pathology practices in various hospital settings [15, 16] and within larger healthcare networks [17, 18] have begun to deploy DP for primary diagnosis. Hence, it is imperative that the appropriate DP displays be used when implementing a DP system, and that the performance of these displays are regularly monitored. To date, there are no guidelines available recognized by the scientific community relating to DP that standardize the minimum test duration time for displays. In this work, we uniformly examined easily verifiable parameters in different categories of monitors at a time technically applicable in everyday life. Herein, we share our experience undertaking a comprehensive “stress test” on four commercial models of DP displays of different quality, monitoring their performance over an extended period. This permitted us to identify which display parameters are most susceptible to aging-related degradation that may impact accuracy and provide recommendations on how to address this technical challenge.

## Material and Methods

Four brand new displays with different technical specifications were selected for testing, including three MG monitors (Barco MDPC-8127, Barco MDRC-8132, and LG 32HL512D) and a non-medical grade device (Samsung Odyssey G55T). For simplicity, they will be referred anonymously to as display 1, display 2, display 3, and display 4. As previously mentioned, MG displays are specifically designed for healthcare use and to meet regulatory standards, such as the CE medical mark, FDA approval [19], and certification from other regulating authorities. The specifications of the selected displays are summarized in Table 1.

**Table 1:** Technical specifications and characteristics of the displays employed

	**Display 1**	**Display 2**	**Display 3**	**Display 4**
Model	Barco MDPC-8127	Barco MDRC-8132	LG 32HL512D	Samsung Odyssey G55T
Display size	27”	32”	31.5”	34”
h × v size (cm × cm)	56.9 × 33.5	70.848 × 39.852	69.73 × 39.22	79.722 × 33.372
Display type	IPS LCD with LED backlight	IPS LCD	Nano IPS LCD with LED backlight	VA LCD
Max resolution	3840 × 2160, 120 Hz	3840 × 2160, 60 Hz	3840 × 2160, 60 Hz	3440 × 1440, 165 Hz
Aspect ratio	16–9	16–9	16–9	21–9
Pixel pitch (mm × mm)	0.148 × 0.155	0.1845 × 0.1845	0.18159 × 0.18159	0.23175 × 0.23175
Flat/curved	Flat	Flat	Flat	Curved, 1000R
Brightness (cd/m^2^)	450 DICOM calibrated 850 maximum	300 DICOM calibrated 500 maximum	450 typ, 360 min, 250 clinical mode, 350 diagnostics mode	250 typ, 200 min
Contrast ratio	1000–1	1000–1	1300–1 typ static, 5 M dynamic	2500–1 typ static, Mega ∞ DCR dynamic
HDR	N-D	N-D	HDR10	HDR10
Colors	10 bit, 1.07 B	30 bit (10 × 3, 1.07 B)	10 bit (8bit + FRC), 1.07 B	8 bit, 16.7 M
Viewing angle	178°	170°	178°	178°
Color gamma NTSC	115% (typical)	80.3%	N-D	72%
Color gamma sRGB	132% (typical)	112%	N-D	N-D
Color gamma DCI-P3	105% (typical)	N-D	98% (CIE1976)	N-D
sRGB delta E2000 (typical)	< 1 average, < 3 maximum	< 3 average, < 5 maximum	< 5	N-D
Response time	8 ms nominal	9.8 ms nominal	14 ms off-settings, 5 ms faster settings	1 ms (MPRT)
Display protection	Antiglare coating	N-D	Antiglare coating, 3H	N-D
Display cost	$$$$	$$$	$$	$

**Fig. 1:**
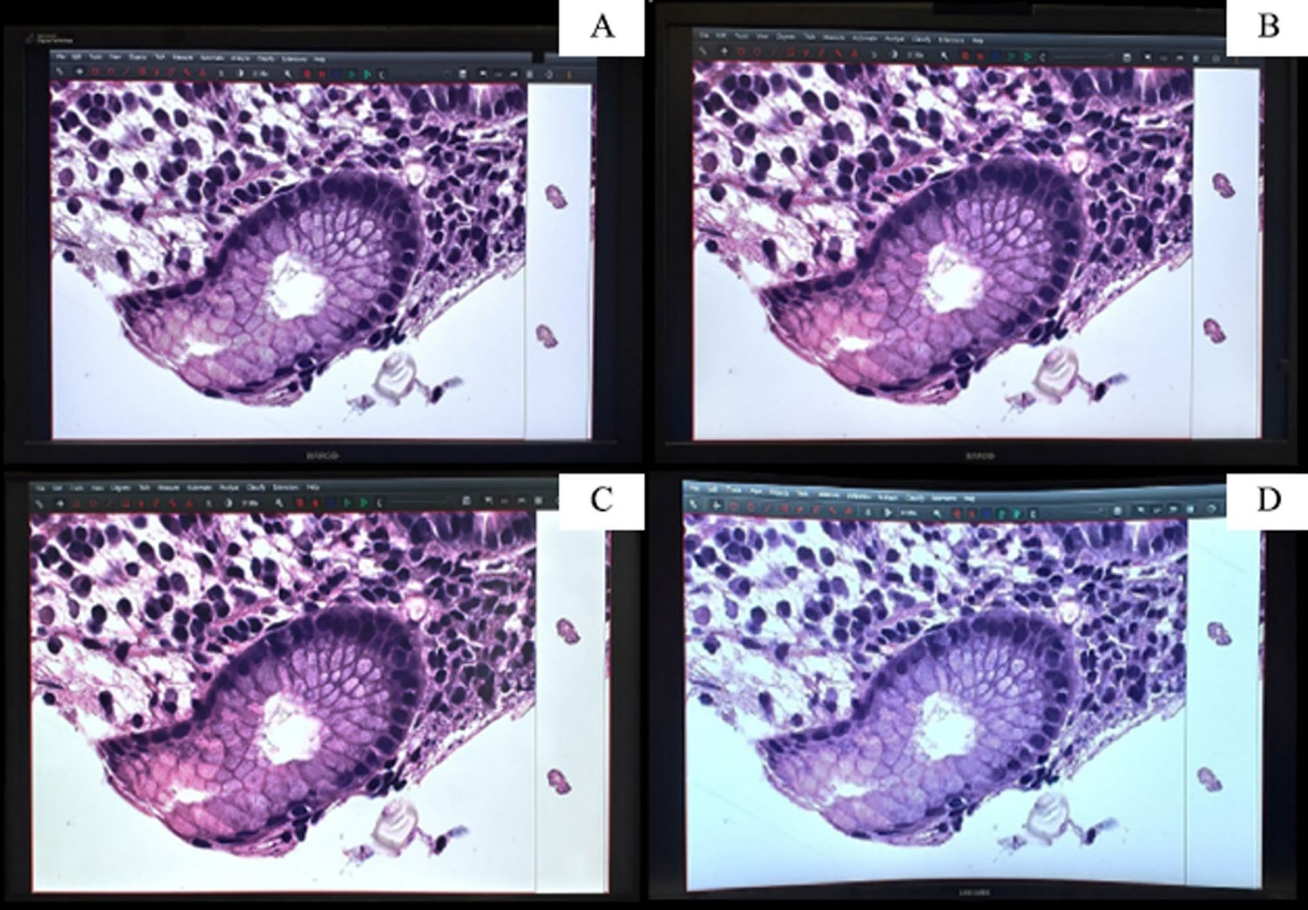
Frontal view of displays: BARCO 8127 (**A**), BARCO 8132 (**B**), LG (**C**), and Samsung (**D**)

All four displays were set up in the same room under identical conditions (Fig. 1). After formal baseline calibration, several test patterns from the American Association of Physicists in Medicine (AAPM) [20] were used to assess various performance metrics, including luminance, contrast ratio, and display uniformity. Although initially used for qualitative analysis in the TG18 report, some of these patterns were applied here for quantitative evaluations. The same person performed all assessments under the same conditions to minimize operator bias.

### Test Conditions

All assessments were conducted under both artificial and natural lighting to detect any variations in display performance. No significant differences were observed between the two lighting conditions, and the effects of artificial lighting were considered negligible. The ambient lighting was not considered for the purpose of the stress test, and all the displays were tested in the same conditions. Over 1 month, displays were tested by continuously playing DP videos for 8 h per day, simulating pathologist routine use. Data were collected daily and analyzed using a temporal averaging method to detect any trends in display performance over time. The decision to conduct a month of measurements is suitable for our designed stress test, and it was made to obtain indications within a reasonable time frame that aligns with the continuous evolution of displays on the market. These technologies constantly evolve, with new products, software, firmware, and driver updates available every 6 months to a year.

**Fig. 2:**
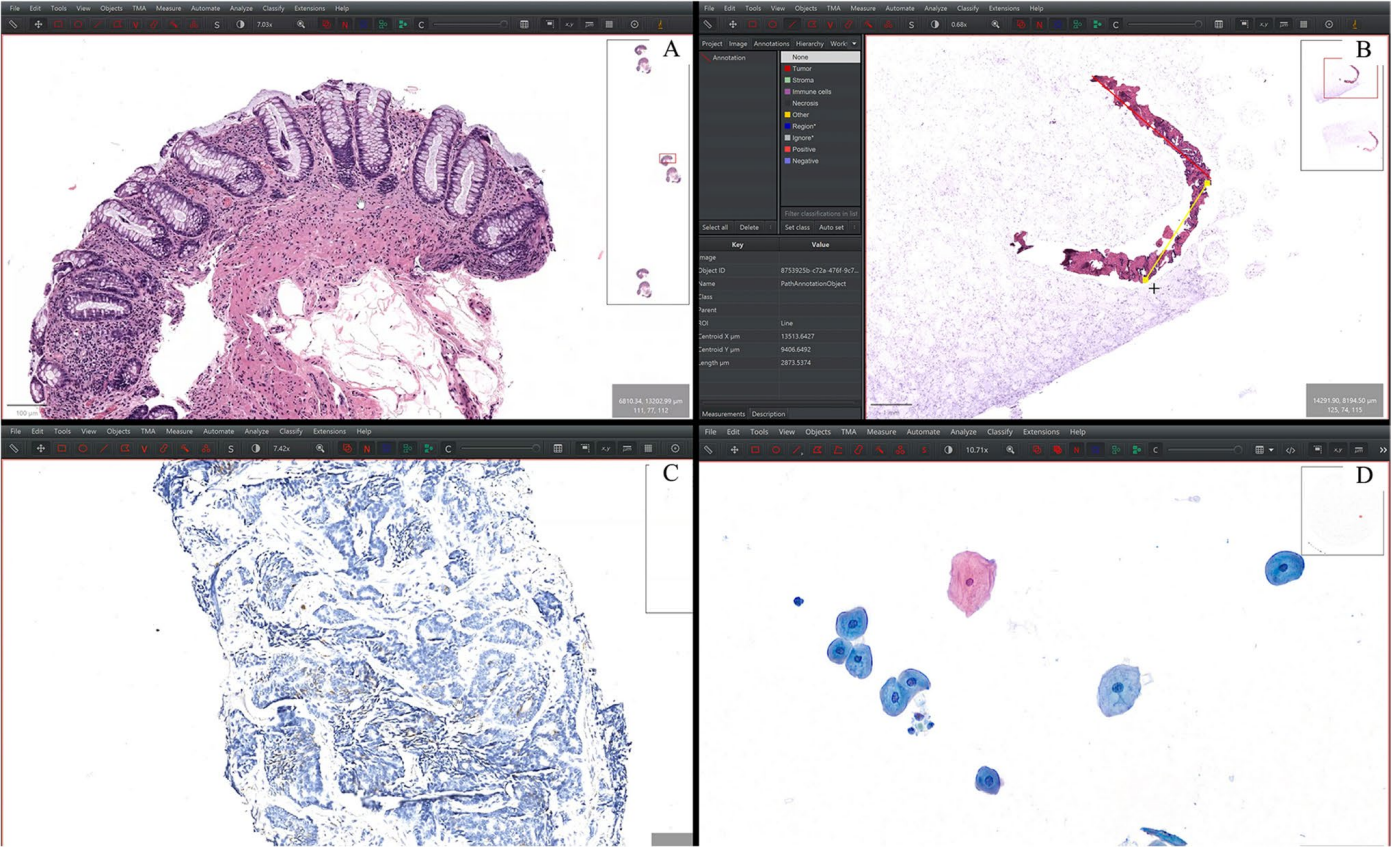
Four screenshots from the recorded video showing the WSI-viewing software (QuPath) during the examination of an H&E-stained colon biopsy (**A**), an H&E-stained prostate biopsy (**B**), an IHC-stained prostate biopsy (**C**), and a Papanicolaou-stained liquid-based urine cytology slide (**D**). Abbreviations: H&E, hematoxylin and eosin; IHC, immunohistochemistry; WSI, whole-slide image

Over two consecutive days, during routine sign-out by a pathologist, the computer monitor displaying whole-slide images (WSIs) was recorded using ffmpeg (https://ffmpeg.org) at full resolution and full-color depth using standard H.264 compression. The final combined recording spanned 8 h and 11 min (Fig. 2). WSIs were viewed using QuPath [21] v0.5.0 and included mainly hematoxylin and eosin (H&E) slides, constituting 69.4% of the global viewing time. Additionally, the evaluation encompassed Papanicolaou-stained cytology slides (24.4% of total time) and diaminobenzidine immunohistochemistry slides (6.2% of total time).

### Technical Specification Measurements

#### Luminance

Luminance, especially maximum luminance (*L*_max_), refers to the brightness a display can achieve when showing its brightest image. For diagnostic purposes, the *L*_max_ setting is often adjusted slightly below the maximum to optimize performance. Medical displays typically present brightness in candelas per square meter (cd/m^2^), while a general purpose displays may show this parameter as a percentage. Since luminance decreases over time with use, regular monitoring is essential. The TG18-LN18 pattern was used to measure the peak luminance of displays employing the Gossen Mavolux 5032B device.

#### Luminance Uniformity

Luminance uniformity measures the consistency of brightness across the entire display. Variations can arise from uneven backlighting or aging of display components, potentially affecting image quality. Common irregularities, such as small, irregularly shaped areas of uneven brightness, may require qualitative evaluation due to their variability. Patterns TG18-UN10 and TG18-UN80 are used to visually inspect uniformity. For more detailed analysis, TG18-UNL10 and TG18-UNL80 patterns are used alongside uniform images, mimicking H&E staining to assess uniformity which is important for pathology (Figure S1). Fifteen points across the display were measured to calculate uniformity, rather than the typical five recommended in AAPM reports [20].

#### Contrast

Contrast is the ratio between the brightest and darkest levels a display can produce, which is crucial for detecting subtle details in images. The contrast ratio is calculated using *L*_max_ and minimum luminance (*L*_min_), with *L*_min_ measured using the TG18-LN01 pattern.

#### Spatial Resolution

Spatial resolution evaluates how well a display can render fine details, which is particularly important in pathology where small features must be accurately visualized. The TG18-QC pattern was used to assess spatial and contrast resolution and to detect any distortion. The TG18-AD pattern was employed to gauge the display’s ability to reproduce fine, high-frequency details.

#### Veiling Glare and Contrast Sensitivity

Veiling glare, caused by light scattering across the display, reduces image clarity in darker areas. Displays with low veiling glare are essential for detecting subtle contrasts in DP images. The TG18-GVN pattern was used to count visible circles at the center of the display, providing a measure of the display’s low-contrast performance. The TG18-BR pattern assessed the monitor’s ability to reproduce high-contrast details by counting the largest squares lacking visible checkerboard patterns.

#### Noise and Small Object Visibility

Noise in a display refers to random patterns that can obscure small, low-contrast details. In DP, these could be diagnostically important features. The TG18-AFC pattern was used to evaluate noise levels and the display’s contrast sensitivity, with particular attention to small objects near the screen edges.

#### Color Sensitivity and Accuracy

Accurate color reproduction is critical in DP, especially for distinguishing H&E stains. Three test images were used to assess the monitors’ color sensitivity. The first two images featured rows of squares with increasing differences in either blue (for hematoxylin) or red (for eosin), simulating stain variations (Figure S2). The number of distinct squares visible in each image was recorded. The third image, based on the point-of-use quality assurance (POUQA) tool [22], tested the display’s ability to show low-contrast color details.

#### Automatic Cell Counting

Differences in technical specifications and display aging can affect how details, such as cells, are rendered in DP. To investigate this, images from the same video frame were captured across different displays and analyzed using an automated script for cell counting based on size, roundness, and color. This test helped assess whether aging and display performance impacted image accuracy.

## Results

### Spatial Resolution

Daily checks using the TG18-QC pattern showed no geometric distortions or resolution losses across all displays. This consistency is essential for DP, where maintaining geometric accuracy and resolution is critical for interpreting fine structures like cellular borders and nuclear details. Any distortion could significantly affect diagnostic accuracy.

### Maximum Luminance and Contrast

Display 1 showed the highest maximum luminance, reaching [440 ± 4] cd/m^2^, followed by display 3 [337 ± 4] cd/m^2^, display 4 [311 ± 2] cd/m^2^, and display 2 with the lowest brightness at [256 ± 2] cd/m^2^. Fluctuations in luminance were minimal, with percentage differences ranging from 2.2 to 4.2% across the displays, indicating stable brightness levels essential for consistent diagnostic image quality. In terms of contrast, display 4 performed a contrast ratio of [3109 ± 18]:1, which may be due to its curvature, exceeding the manufacturer’s declaration and representing a point of investigation. Display 1 maintained a contrast ratio of [988 ± 9]:1, close to its expected 1000:1. However, displays 2 and 3 underperformed with contrast ratios of [855 ± 7]:1 and [843 ± 11]:1, respectively, below their specified values. The variations, in contrast, were mainly due to changes in peak luminance, as low luminance levels remained constant throughout the study.

### Luminance Uniformity

Testing with the TG-UNL10 pattern showed comparable performance for displays 1, 2, and 3, although statistically significant differences were observed. Display 1 [15.9 ± 3.6] and display 2 [17.4 ± 2.3] showed a significant difference (*t* = 2.38, *p* = 0.02), and display 1 outperformed display 3 [18.6 ± 3.8] with more pronounced variation (*t* = 3.53, *p* = 0.0007). Over time, uniformity slightly changed by 3.8%, 2.6%, and 5.3% for displays 1, 2, and 3, respectively. Display 4 [38.6 ± 2.9] showed worse uniformity and a change of 4.9%. In the TG-UNL80 pattern test, display 1 showed uniformity [8.0 ± 1.5], which was significantly better than the other displays. Display 3 [13.9 ± 1.7] slightly outperformed display 2 [16.1 ± 1.4], although their performance was close. Display 4 showed a uniformity of [21.4 ± 2.2], with a 2.5% variation over time. Using the hematoxylin-like pattern, display 1 showed the best performance [9.4 ± 0.9] with minimal variation. Displays 2 with [22.6 ± 0.7] and 3 with [22.5 ± 1.8] were statistically similar, but display 3 had a uniformity variation of 4.2%. Display 4 showed a value of [33.3 ± 1.2], with a 3.1% variation over time. For the eosin-like color pattern, display 1 led with [9.9 ± 0.8], followed by display 3 [20.5 ± 1.6], display 2 [22.9 ± 0.8], and then display 4 [30.0 ± 0.6].

### Color Contrast and Sensitivity

Using the hematoxylin contrast test, displays 1 and 3 allowed clear visualization of squares in the lowest contrast lines [48 ± 1] and [46 ± 1] squares, respectively, while display 2 showed [41 ± 1] squares. Display 4 had difficulty detecting lower contrast squares [35 ± 2]. In the eosin contrast test, display 1 excelled with [48 ± 1] squares visible, while displays 2, 3, and 4 showed [40 ± 1], [30 ± 1], and [34 ± 2] squares, respectively. In the POUQA-inspired color test, all displays met the minimum contrast requirements. Display 3 performed the best, detecting the smallest numbers in all sectors down to 8 pixels. Display 1 detected numbers down to 12 pixels in most sectors and display 2 down to 14 pixels. Display 4 lagged, detecting numbers only down to 14 pixels in the final sector.

### Veiling Glare and Contrast Details

The TG18-GVN pattern test showed that displays 1, 2, and 3 could display all five low-contrast circles clearly. Display 4 only managed four circles, but still met the minimum requirement. In the TG18-AD test, displays 1, 2, and 3 identified patterns in 47 out of 49 quadrants, while display 4 managed only 35 quadrants, indicating weaker contrast resolution. The TG18-BR pattern showed improvement over time, likely due to user familiarity with detecting contrasts. Display 1 performed well, missing only the lowest two contrast panels. Displays 2 and 3 performed similarly, while display 4 missed more targets, indicating a lower ability to detect fine contrast details.

### Noise and Small Object Visibility

In the TG-18 AFC pattern test, display 1 detected all 16 points, indicating strong performance in detecting fine details. Display 3 initially missed one point but improved after sharpness adjustments. The sharpness was set to maximum for uniformity and to reduce variability or not introduce bias. Display 2 consistently detected 15 points, while display 4 improved from 13 to 14 points after adjustments but still fell short of the others. These results underscore the importance of high-quality displays for detecting minute microscopic details in pathology images.

### Automatic Cell Counting

Using the automatic cell counting test, displays 1 and 2 performed similarly, with cell counts of [135 ± 12] and [137 ± 14], respectively. Display 3 was close, with a slightly lower value of [130 ± 14], while display 4 underperformed with [122 ± 12], suggesting it may miss subtle features critical for accurate cell detection and counting. Display 4 was statistically significantly different from the other displays (*p* < 0.05).

A summary of the parameters and values measured during the entire 1-month stress test study period is presented in Table 2.

**Table 2:**
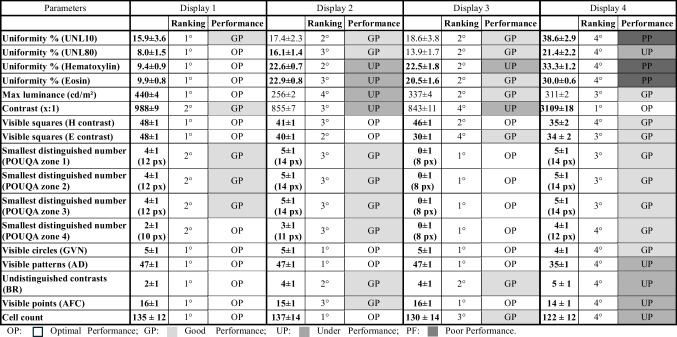
Performances of the different displays among the investigated parameters

## Discussion

Digital pathology remains disruptive, especially as new applications become integrated and validated into daily practice [23]. Displays comprise a vital component of a DP system and are used to view digital slides akin to viewing glass slides with a conventional light microscope. As such, it is important that displays be validated for diagnostic clinical work. However, the pathology literature is limited in this regard. In fact, only a few studies address the performance of computer monitors in various fields [24]. Thus, the present study contributes to this important body of literature, in which we assessed various parameters in monitors when utilized for DP under stress conditions. We recorded a video of the routine sign-out of a pathologist that closely matched typical working conditions. Since recommendations [25] suggest using one dedicated monitor to view WSIs and another to interact with the laboratory information system (LIS), the video recording in our study included only the WSI-viewing monitor. The recording included pauses in which the monitor was not actively used, e.g., while typing the pathology report on the other monitor. All monitors were set up for the same WSI fields and located in identical room conditions to reduce internal or external biases. We chose to initially limit the recording time to 8 h per day, with an overnight washout, to closely simulate a pathologist’s routine workflow.

Our stress test revealed that the monitors tested may perform and age differently reflecting their varied quality and technical specifications. Several reasons may be responsible for display aging, including wearing out of phosphors backlights, liquid crystal breakdown, and corrosion of electric contacts or optical materials, among others [26, 27]. These considerations notwithstanding, the reduction of a display’s performance relative to its aging may affect the ability to read WSIs, ultimately influencing diagnostic accuracy [28]. Overall, all the monitors in this study performed well for most of the investigated parameters, with a slightly better outcome observed for the MG displays. Thus, while MG displays are often recommended, COTS monitors might still be satisfactory for handling routine pathology tasks [29]. It is worth underlining that this study was not meant to make specific recommendations for display purchases, as that would require a more detailed cost–benefit analysis.

Among the various specifications, monitors 1, 2, and 3 maintained acceptable uniformity below the 20% threshold for TG18-UNL10 and TG18-UNL80 patterns. However, uniformity values varied for some monitors during these stress tests, suggesting the need for periodic quality checks to prevent degradation. In DP, there is limited research on how luminance uniformity might affect diagnosis, but the visible variations in this study potentially highlight its importance. When testing with stain-like images, only display 1 maintained the standard below 20% uniformity, suggesting that extending uniformity evaluations to colored images is useful for performance comparison between displays.

As for the other parameters evaluated, no significant change in maximum luminance was observed. A longer stress test could likely provide more useful information. Previous studies indicate that extended use can reduce luminance [30] potentially compromising diagnostic quality [28]. Pathology guidelines suggest a peak luminance of 300 cd/m^2^ for diagnostic displays [31]. Lower luminance might have marginal effects on diagnostic performance, even if high luminance might be preferred by pathologists [32]. Similarly, contrast levels showed minimal variation during testing. Whereas contrast is considered an important factor in DP, its influence on display preference remains debated. While some studies indicate no clear preference for higher contrast [32], others suggest it can affect visual comfort when combined with increased brightness [33]. A contrast ratio of 1000:1 is generally recommended, and such a goal was met by two of the investigated displays 1 and 4. Finally, the resolution did not show visible degradation during the stress test among the different devices, which was assumed to be due to the short test duration. Although higher resolution can lead to faster workflow [34] and higher pathologist preference [32], it should not be a defined standard for DP diagnostic accuracy [35, 36].

This work carries some intrinsic limitations. First, the ambient lighting during our experiments was relatively low, with illuminance levels around 20–100 lx. This is lower than the typical illuminance levels found in a bright office space used by pathologists, which are generally around 300–500 lx. The effects of strong external illuminance, such as those typical of a pathologist’s working environment, were not tested. Additionally, the sensitivity instrument could be affected by luminance level, possibly causing it to miss tiny variations in dark point luminance that could affect parameters like contrast ratio. Another point to consider is that while the three MG displays were calibrated on the same luminance response function, this was not true for the COTS, which was left uncalibrated in its native state. Therefore, it is unknown if the COTS could perform better if calibrated like the other displays. It could also be inferred that only a few devices were tested and that a 1-month analysis period is too short to highlight the impact of real-world differences. However, the comprehensive evaluation of many aging-related factors, encompassing parameters potentially impacting the final diagnostic report, strengthens the value of our stress test design. Moreover, it is important to recognize that our work is a pioneering study in this field, as correlations between display characteristics and digital pathology outcomes are still underdeveloped. While AAPM thresholds provide a useful starting point, further research is warranted to address similar issues. This will help build a solid collection of articles that can serve as key references on the topic. Lastly, extensive subjective participation from pathologists and trainees was not leveraged, and this could have contributed to evaluating the importance of different parameters. Evidence of partial changes in the displays during the monthly tests leads us to believe that a quality assurance program with automatic tests could be strategic. Introducing common test pattern images into the monitors automatically or via remote self-calibration systems could be important for maintaining uniformity in reading DP images and reducing possible biases or errors. Based on the minimum appreciable variability observed during the monthly stress test period, a quality program with automatic self-calibration and verification systems on a monthly basis, along with half-yearly verification of specific parameters that influence image quality, could contribute to greater system stability.

## Conclusion

In our stress test designed to simulate DP sign out in practice, we have demonstrated that computer displays employed for DP tasks vary in quality related to their specifications, with MG monitors generally outperforming a COTS display. While some parameters, including maximum luminance, contrast, and resolution, remained stable, others, such as luminance uniformity, showed greater variability. Thus, these data advocate for routine quality checks of displays to avoid aging-related effects that could potentially negatively impact DP diagnoses. Further research regarding displays in DP systems and practical guidelines on how best to continually monitor their performance is recommended.

## Supplementary Information

Below is the link to the electronic supplementary material.

Supplementary file1 (JPG 71 KB)

Supplementary file2 (JPG 86 KB)

## Data Availability

All data generated or analysed during this study are included in this published article.
