# Supplementary figures and images for: Digital Pathology Displays Under Pressure: Benchmarking Performance Across Market Grades
Source: J Imaging Inform Med. 2025 Feb 26;38(6):4090–9. doi: 10.1007/s10278-025-01452-3 (PMC12701151; doi:10.1007/s10278-025-01452-3)

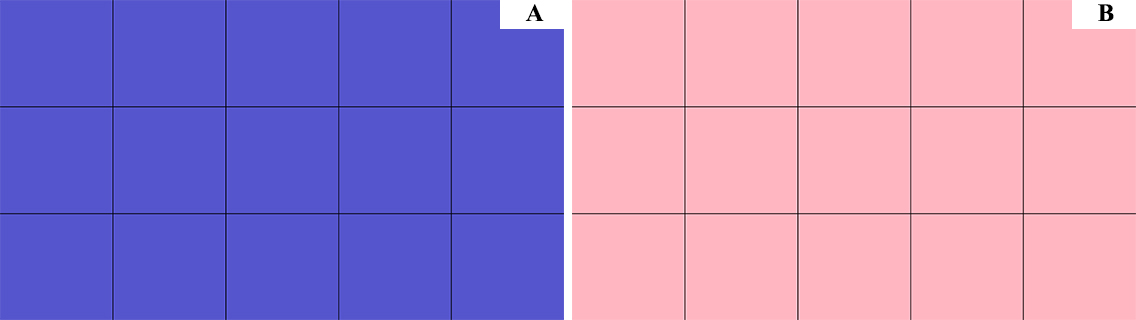

Supplement: Supplementary file 1 — Supplementary file1 (JPG 71 KB) [file 10278_2025_1452_MOESM1_ESM.jpg]

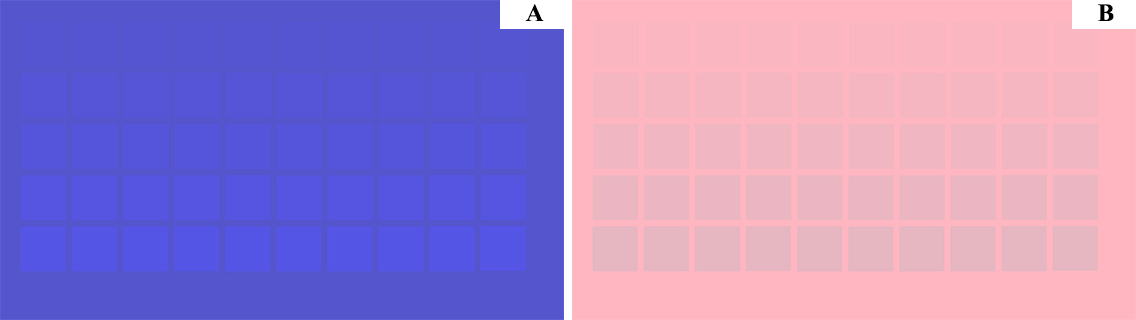

Supplement: Supplementary file 2 — Supplementary file2 (JPG 86 KB) [file 10278_2025_1452_MOESM2_ESM.jpg]
